# Analysis of Tyre Pyrolysis Oil as Potential Diesel Fuel Blend with Focus on Swelling Behaviour of Nitrile-Butadiene Rubber

**DOI:** 10.3390/polym17223016

**Published:** 2025-11-13

**Authors:** Steffen Seitz, Tobias Förster, Sebastian Eibl

**Affiliations:** 1 Institute of Mechanics, Faculty of Aerospace Engineering, University of the Bundeswehr Munich, Werner-Heisenberg-Weg 39, 85579 Neubiberg, Germany; 2 Bundeswehr Research Institute for Materials, Fuels and Lubricants (WIWeB), Institutsweg 1, 85435 Erding, Germany; tobias1foerster@bundeswehr.org (T.F.); sebastianeibl@bundeswehr.org (S.E.)

**Keywords:** nitrile-butadiene rubber, tyre pyrolysis oil, chemical analysis, swelling behaviour, mechanical testing

## Abstract

This study examines the swelling behaviour of nitrile-butadiene rubber (NBR) when interacting with tyre pyrolysis oils (TPO), with a focus on the chemical composition of TPO and their interaction with rubber matrices. Initially, a comparative analysis with conventional diesel fuel (DF) was performed using advanced analytical techniques, including two-dimensional gas chromatography coupled to mass spectrometry (2D-GC/MS), infrared (IR) spectroscopy, and nuclear magnetic resonance (^1^H-NMR) spectroscopy. The analysis revealed that TPO contains a significantly higher proportion of aromatic hydrocarbons than DF, along with unsaturated and oxygen-containing compounds not present in DF. Based on these compositional differences, blends of TPO and DF were formulated and evaluated for their suitability as liquid energy carriers according to the specifications of DF. In principle, blends with an addition of up to 5 vol% TPO in DF are technically suitable for use as fuel. Subsequently, the sorption behaviour of TPO, DF, and their blends in NBR was investigated. The swelling potential was determined based on mass, density, and volume, and the changes in the hardness and tensile strength of NBR were recorded. The results demonstrate that TPO induces pronounced swelling in NBR, as evidenced by a marked increase in mass uptake and volume expansion. A linear increase was observed between the degree of swelling and the increasing TPO content in the blends. Mechanical property assessments revealed a corresponding decrease in the hardness and tensile strength of NBR upon exposure to TPO, with the most severe effects associated with neat TPO. This work provides a comprehensive assessment of TPO as a potential blend component for DF. It highlights the need for careful consideration of material compatibility in practical applications.

## 1. Introduction

Around 3.4 million tons of tyre waste are generated across Europe every year. Processes for recycling tyres include combustion, which is predominantly used in the cement industry and material reuse, for which the plastic components have to be separated. Due to the complex composition of tyres, the challenge is to achieve complete separation of the components. The composition of tyres varies depending on their application. According to manufacturers, tyres typically consist of 41% rubbers, 30% fillers, 15% steel and textiles, 6% plasticizers, 6% vulcanization reagents, and 2% antioxidants [1]. The pyrolysis process is making the complete recycling of used tyres increasingly important. Textiles and steel can be separated from the rubber and recycled to produce new tyres. During pyrolysis, polymer chains in the tyre granulate are depolymerized under inert conditions to produce gas, oil, and coke residues [2,3,4]. The quantity of pyrolysis products depends on the type of tyres used and the process parameters [5,6,7]. T. Williams has evaluated various reactor types that were tested to preferably gain tyre pyrolysis oil (TPO) [8]. Pyrolysis gas is mostly used as an energy source and the solid residue can be reprocessed as recycled carbon black in tyres [3,9].

The individual pyrolysis mechanisms have been described by several authors for the elastomer types of natural rubber (NR), styrene-butadiene rubber (SBR) and butadiene rubber (BR) [10,11,12,13,14,15]. Due to the complexity of the tyre composition, further studies were carried out for corresponding elastomer mixtures [16,17,18,19,20]. In general, the polymer chains undergo depolymerization, disproportionation reactions or β-cleavages and are pyrolyzed into smaller molecules such as monomers, dimers, and oligomers. For example, NR predominantly forms isoprene and dipentene and BR produces butadiene, vinyl cyclohexene, and dipentene [10,15,21]. Diels–Alder reactions, addition reactions, cyclisation, aromatisation, and intramolecular hydrogen transfer convert the primary products into benzene, indene, and naphthalene with alkyl and alkenyl substituents [11,15,19]. SBR pyrolyzes mainly to styrene and methyl styrene and reacts via Diels–Alder reaction to alkylbenzenes or diaromatics like biphenyl, and with the BR and NR products to alkyl naphthalenes [12,13,14,16]. The oil component can be used as a fuel [22,23]. TPO is regarded as a possible blend for diesel fuel (DF) due to its similar composition [24]. Pyrolysis thus contributes to more sustainable tyre recycling and the conservation of fossil resources.

When TPO is used as a liquid energy carrier in civil or military transport vehicles, it is necessary to ensure operational availability and that TPO is compatible with the system. Compatibility with the elastomers must hence be analyzed. The resistance of sealing materials depends on the chemical composition of the liquid medium [25,26]. It is, therefore, crucial to determine the chemical composition of TPO when estimating its diffusion behaviour. This can be observed through swelling, extraction of additives, and changes in the mechanical properties of the elastomer [27,28,29]. Nitrile-butadiene rubber (NBR) is an elastomer widely utilized in the automotive and aerospace industries for applications such as seals, hoses, and tank linings. Its utilization is primarily attributed to its excellent resistance to non-polar liquids, including fuels, as well as its favourable mechanical and ageing properties [30]. With an increasing acrylonitrile content in the polymer, the polarity of the NBR increases, leading to better resistance to non-polar liquids [31,32].

This study aims to investigate the potential use of TPO as a replacement or blend for DF and to understand TPO’s diffusion behaviour in the established sealing material NBR. On the one hand, the focus is on comparing the chemical composition of DF and TPO and determining the characteristic properties of TPO-DF blends according to DIN EN590 [33]. On the other hand, the work presents compatibility tests of the liquids with NBR with different acrylonitrile content to estimate its swelling potential and resulting influence on mechanical properties. In addition, the temperature dependence of the diffusion process of neat TPO and DF is investigated. The results contribute to an understanding of the sorption behaviour of TPO in contact with NBR and promote its potential use as a liquid energy source.

## 2. Materials

Polymer Service Merseburg (Merseburg, Germany) produced sulphur cross-linked nitrile-butadiene rubber (NBR) samples with an acrylonitrile content of 18 wt%, 28 wt%, and 39 wt% in the NB-copolymer. In this study, these are abbreviated as NBR18, NBR28, and NBR39. The exact formulations are shown in Table 1. The NBR samples contained additives, including DPHP and 6-PPD, which served as plasticizers and antioxidants. The cross-linking of the elastomer required zinc oxide, stearic acid, and sulphur, while N-Cyclohexyl-2-benzothiazolylsulfenamide (CBS) and Tetramethyl thiuram monosulfide (TMTM-80) acted as accelerators for the vulcanisation process. S2 Tension rods were punched out of the NBR sheets [34]. These were split to a thickness of 1 mm using a band knife splitting machine NAF470 from Fortuna GmbH (Weil der Stadt, Germany).

To produce tyre pyrolysis oil (TPO), Pyrum Innovations AG from Dillingen/Saar (Germany) provided thermolysis oil from a mixture of truck, car, and bicycle tyres. The TPO was vacuum filtered for further use. Blends with the pyrolysis oil were created using a commercially available diesel fuel (DF) according to DIN EN 590. The mixing ratio was 1 vol%, 5 vol%, and 10 vol% of TPO in DF, abbreviated as DF99TPO1, DF95TPO5, and DF90TPO10.

## 3. Experiment

### 3.1. Analysis of Fuels

The liquids were tested using the A-test according to DIN EN 590 (2022-05) for DF [33]. The individual test methods and the standards used are shown in Table 2. In addition, the total acid number (TAN) was determined according to ASTM D664 [35]. A potentiometric titration with potassium hydroxide (KOH) was used for this determination. TAN represents the mass of KOH, in milligrams, required to neutralize one gram of the sample. Using the concentration (*c*) of the KOH solution, the sample mass (*m_s_*), and the titration volumes of the blank (*V_B_*) and the sample (*V_S_*), the TAN can be calculated according to Equation (1).

(1) $$TAN=\frac{56.1\times c(V_{S}-V_{B})}{m_{S}}$$

The iodine value is calculated in accordance with DIN EN ISO 3961 [36]. In this method, iodine chloride reacts with carbon–carbon double bonds, followed by the addition of potassium iodide to release iodine, which is subsequently titrated with sodium thiosulfate. The resulting iodine number is obtained from Equation (2), which relates the concentration of the sodium thiosulfate solution (*c*), the titration volume of the blank (*V_B_*) and for the samples (*V_S_*), and the mass of fuel sample (*m_s_*).

(2) $$Iodine\;value=\frac{12.69\times c(V_{B}-V_{S})}{m_{S}}$$

**Table 2:** Selected parameters from DIN EN 590 with standards used.

Parameter	Norm
Viscosity	ASTM D7042-21a [37]
Density	
Polyaromatic content	DIN EN 12916 [38]
Flash point	DIN EN ISO 2719 [39]
HFRR ^1^-test	DIN EN ISO 12156-1 [40]
CFPP ^2^	DIN EN 116 [41]
Cupper corrosion	DIN EN ISO 2160 [42]
Oxidation stability	DIN EN 16091 [43]
Water content	DIN 51777-2 [44]
Carbon residue	DIN EN ISO 10370 [45]
Ash	DIN EN ISO 6245 [46]
Sulphur content	DIN EN ISO 20884 [47]
Distillation process	DIN EN ISO 3405 [48]

^1^ HFRR (high frequency reciprocating rig); ^2^ CFPP (cold filter plugging point).

Two-dimensional gas chromatography coupled to mass spectrometry (2D-GC/MS) analyses were performed with a Shimadzu GC-2010 Plus gas chromatograph equipped with a ZOEX ZX1 modulator and a Shimadzu GCMS-QP2010 Ultra mass spectrometer operated in electron ionization mode. The column combination used was a 30 m Restek Txi-1ms (0.25 mm; 0.25 µm film) with 3 m SGE Analytical Science BPX 50 (0.15 mm; 0.15 µm film). Measurements were performed in 1:10 split mode with helium as a carrier gas and a modulation time of 8 s. The oven temperature was maintained at 50 °C for 3 min, before heating to 320 °C at a constant heating rate of 5 °C min^−1^, where it was held for 2 min.

In addition, 10 mL TPO was added to a HyperSep silica stationary solid phase extraction (SPE) column from Thermo Scientific and washed with 20 mL heptane and 10 mL acetone. The polar acetone fraction was analyzed by GC/MS using an HP5 column at 50 °C for 2 min and a 10 °C min^−1^ heated to 300 °C and held for 2 min.

^1^H-Nuclear magnetic resonance (^1^H-NMR) analyses were performed with a Spinsolve 80 Carbon from Magritek in Powerscan mode at 80 MHz and analyzed with MestReNova software. Octamethyl silane was added to TPO as reference.

Fourier transform infrared (FTIR) spectra were recorded with a Bruker Tensor 27 spectrometer, which was equipped with a 50 µm zinc selenid cuvette at a spectral resolution of 4 cm^−1^. The spectra were averaged over 32 scans in a wavenumber range between 4000 cm^−1^ and 500 cm^−1^. The spectra were baseline corrected and normalized to the band intensity at 2725 cm^−1^.

### 3.2. Sorption Experiments

For preparation, the volatile components such as plasticizer DPHP and antioxidant 6-PPD were extracted from the NBR samples in a Soxhlet apparatus for 16 h with acetone. For the sorption tests, the NBR samples were stored in the liquids for 0.5 h, 1 h, 7 h, 24 h, 72 h, and 168 h. The NBR28 and NBR39 samples were additionally stored for 672 h and 2016 h and NBR39 for 4032 h. Additionally NBR18 were kept in TPO and DF at 50 °C and 80 °C. The relative mass change Δ*m_grav_* was calculated using Equation (3), where *m_t_* is the mass after a certain storage time, and *m*_0_ is the initial mass.

(3) $$∆m_{grav}=\frac{\left(m_{t}-m_{o}\right)}{m_{0}}*100wt\%$$

Using Fick’s second diffusion law and the solution for one-dimensional diffusion, Equation (4) can be derived [49]. This Equation uses a sorption curve to determine the specific relative mass changes. The mean diffusion coefficient *D* can be determined by calculating the mass after specific time intervals *m_t_*, the thickness of the NBR samples *h* (1 mm), the equilibrium mass *m*_∞_ of the samples and the number of iteration steps *n*.

(4) $$\frac{{∆m}_{t}}{∆m_{\infty }}=1-\frac{8}{\pi^{2}}\sum_{n=0}^{\infty }\left(\frac{1}{{\left(2n+1\right)}^{2}}exp\left(\frac{{-(2n+1)}^{2}\pi^{2}Dt}{h^{2}}\right)\right)$$

After storage, the samples were immersed in 40/60 gasoline to remove any fuel adhering to the outside and blotted with a paper towel. To determine the density, the samples were weighed in water using the VF 4601 kit from Satorius (Göttingen, Germany) and calculated according to Archimedes’ principle using Equation (5). The volume change in the NBR sample can be determined using Equation (6) with mass and density.

(5) $$\rho_{NBR}=\frac{m_{NBR,Air}\;*\;\rho_{Water}}{m_{NBR,Air}-m_{NBR,Water}}$$

(6) $$∆V=\frac{\left(V_{t}-V_{o}\right)}{V_{0}}*100\;vol\%$$

A Bareiss^®^ digi test II testing machine (Oberdischingen, Germany) was used to determine the micro-Shore-A hardness of the NBR according to DIN ISO 48-4:2021-02 [50]. Tensile tests according to DIN 53504 were carried out to determine the elongation at break and tensile strength using a Zwick A universal testing machine from ZwickRoell GmbH (Ulm, Germany) with a 500 N load cell and optical extensometer with an elongation rate of 0.167 s^−1^ [34].

## 4. Results

### 4.1. Comparison of the Chemical Composition of DF and TPO

#### 4.1.1. GC/MS Analysis

**Figure 1:**
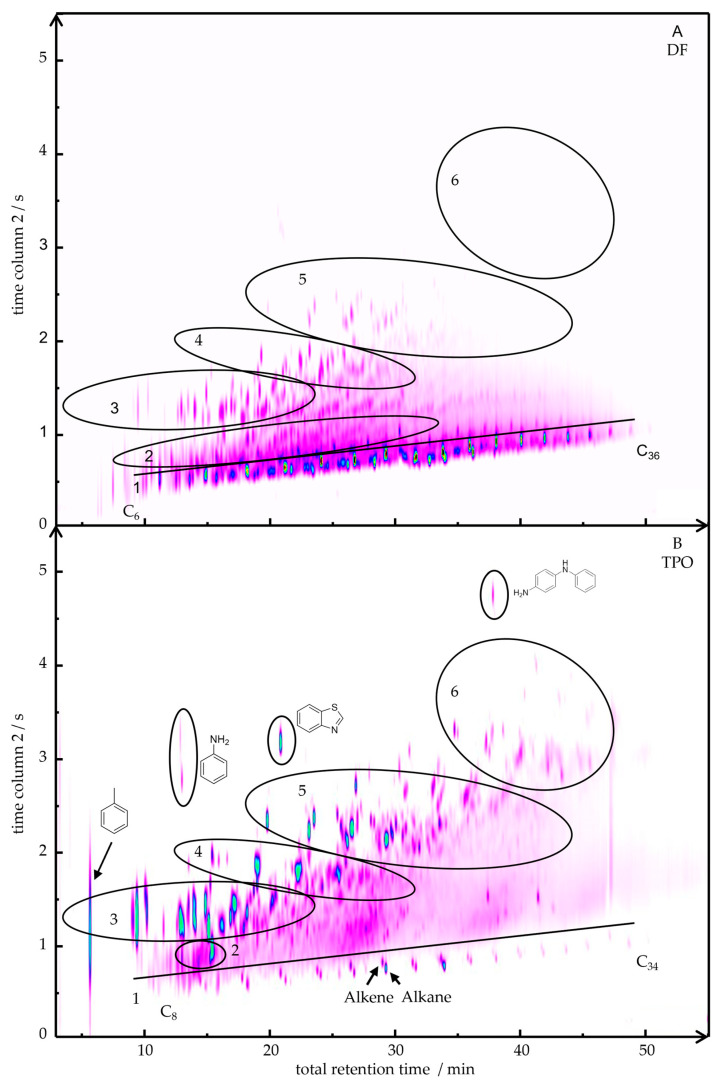
2D-chromatograms of DF (**A**) and TPO (**B**) with marked areas of substance classes: 1 Alkanes and Alkenes; 2 Cyclic alkanes and alkenes, 3 Monoaromatics, 4 Indenes, 5 Diaromatics, 6 Polyaromatics.

Figure 1 presents the two-dimensional gas chromatogram (2D-GC/MS) of DF (Figure 1A) and TPO (Figure 1B). For a semi-quantitative analysis, the areas of the individual peaks from the 2D GC-MS chromatogram were integrated and assigned to a substance class based on the mass spectra. The results of this classification are summarized in Table 3. In region 1 TPO shows a homologous series of alkanes ranging from C_8_ to C_34,_ along with the corresponding mono- and diunsaturated alkenes. In contrast, DF contains a broader range of alkanes (C_6_ to C_36_) with higher degree of structural variability in the branches and higher relative signal intensity (74 area%) compared to TPO (8 area%). Region 2 of the chromatogram reveals cyclic alkanes with methyl and ethyl substituents in DF (9 area%), whereas TPO (7 area%) is characterized by an intense peak of substituted cyclohexene (1-methyl-4-(1-methylethyl)-cyclohexene). Toluene is detectable in TPO at low retention time. Monoaromatic hydrocarbons are detected in region 3 for both DF and TPO. They consist of mono-, di-, tri-, or tetra-substituted methyl- or ethylbenzenes. Prominent examples include 1,3-diemehtylbenzene, 1-ethyl-2-methylbenzene, mesitylene, 1-methyl-4-(1-methylethyl)-benzene as well as with linear and/or branched C_3_- to C_8_-alkylbenzenes such as pentylbenzene. Similar substituents as in the aromatics are identified in region 4 for the various indenes (e.g., 1-methyl-1H-inden; 1,2,3-trimethylindene).

Naphthalenes substituted with mono-, di-, or trimethyl groups (e.g., 1-methylnaphthalene, 1,6-dimethylnaphthalene, 2,3,6-trimethylnaphthalene) and dihydronaphthalene (e.g., 1,2-dihydro-6-methylnaphthalene) can be seen in region 5. In addition, other diaromatic hydrocarbons including, e.g., biphenyl, fluorene, and chamazulene are detected in TPO. The most notable contrast in region 3 to 5 lies in the markedly higher peak intensity of the aromatic compounds in TPO. Polyaromatic hydrocarbons (PAHs) such as anthracene, pyrene, and retene, which are only present in TPO (5 area%), can be seen in range 6. Furthermore, TPO contains nitrogen-, oxygen-, and sulphur-containing compounds (6 area%) with the most intense occurrences of aniline, benzothiazole, and N-phenyl-1,4-benzenediamine, as illustrated in Figure 1.

The polar substances in the TPO were enriched by SPE and analyzed by GC/MS. Nitrogen-containing substances were detected, such as amines, with cyclic and linear hydrocarbons, and anilines, such as diphenyl diamines. Various oxygen-containing substances, such as phenol or stearic acid, palmitic acid, and myristic acid, could be detected in the TPO. The presence of heteroatoms can result from the additives used during tyre manufacturing. These include, for example, vulcanization agents (such as thiazoles, organic peroxides, nitro compounds), antioxidants (amines, phenols), plasticizers (aromatic esters), or processing aids (such as mineral oils and peptizers) [51]. In addition, during tyre usage, oxygen can diffuse into the material, initiating ageing processes such as oxidation. As a result, oxygen-containing compounds may form during pyrolysis [52].

In summary, significant differences in the chemical composition of TPO and DF were detected. DF showed a higher content and variability of linear and branched alkanes and cycloalkanes. TPO indicated linear alkanes and branched alkenes, and the corresponding content of monoaromatic and diaromatic compounds was much higher. Additionally, TPO showed PAHs and nitrogen-, oxygen- and sulphur-containing compounds, which were not detected in DF.

#### 4.1.2. ^1^H-NMR-Spetroscopy

**Figure 2:**
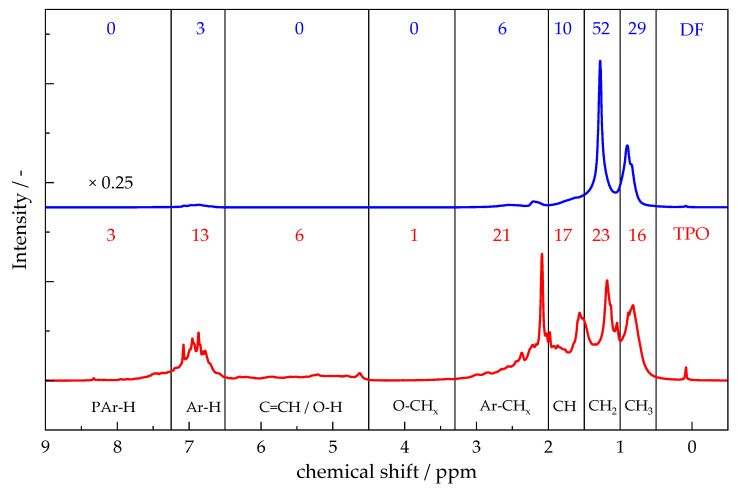
^1^H-NMR spectra of TPO an DF with the corresponding percent of hydrogen atoms in the defined areas. 0.5–1 ppm “CH_3_ aliphatic”; 1–1.5 ppm “CH_2_ aliphatic; 1.5–2 ppm, “CH aliphatic”; 2.0–3.3 ppm “aliphatic bonded to aromatic; 3.3–4.5 ppm “aliphatic bonded to oxygen”; 4.5–6.5 ppm “olefins and hydroxyl”; 6.5–7.25 ppm “monoaromatic”; 7.25–9 ppm “polyaromatic”.

Figure 2 presents the ^1^H-NMR spectra of TPO and DF. The application of this technique facilitates the comparison of functional group distributions in complex liquid mixtures through the quantification of hydrogen environments. The majority of hydrogen atoms in DF and TPO are associated with aliphatic hydrocarbon chains with 91% for DF and 56% for TPO (0.5–3.3 ppm) [53,54,55,56]. These signals can be further separated and attributed to CH_3_– (0.5–1 ppm), CH_2_– (1–1.5 ppm) and CH–groups (1.5–2.0 ppm). Hydrogen in carbon chains adjacent to aromatic rings, like α-methyl carbons, appears between 2.0 ppm and 3.3 ppm and comprises 6% in DF and 21% in TPO [53,57]. A minor signal in TPO between 3.3 ppm and 4.5 ppm indicates a small amount (1%) of hydroxyl groups [54]. TPO also exhibits a signal (4.5 ppm and 6.5 ppm), representing hydrogen atoms bound to unsaturated hydrocarbons or phenolic structures, with a relative intensity of 6% [54,57]. Hydrogen atoms directly attached to monoaromatic rings can be located in the range of 6.5–7.25 ppm, with 3% in DF and more prominently with 13% in TPO. Signals between 7.25 ppm and 9.0 ppm, corresponding to polyaromatic structures, are observed in TPO (3%), as described by Rodríguez et al. [58,59]. The results differ from the 2D-GC/MS analysis, which detected polyaromatic compounds for TPO and Diaromatics for DF.

TPO exhibits a lower proportion of hydrogen in aliphatic carbon chains compared to DF, but a higher proportion of hydrogen atoms directly bound to aromatic rings. The proportion of carbon chains bound to an aromatic ring is also significantly higher in TPO. The presence of polyaromatic and unsaturated structures in TPO, which are absent in DF, highlights the considerable differences in molecular composition between the two substances. In summary, the results correspond to the 2D-GC/MS results.

#### 4.1.3. ATR-FTIR-Spectroscopy

**Figure 3:**
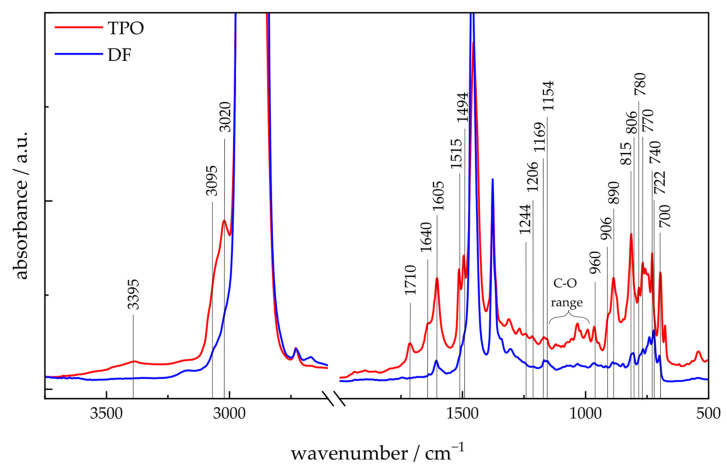
FTIR spectra of TPO and DF with indication of important bands.

Figure 3 presents the ATR-FTIR spectra of TPO and DF. For both fluids the C-H valence vibrations of alkanes are observed between 3000 cm^−1^ and approx. 2700 cm^−1^, and the corresponding deformation vibrations for CH_2_ and CH_3_ are at 1460 cm^−1^ and 1385 cm^−1^, respectively [60,61]. A mixture of iso-alkanes can be assigned in TPO and DF at 1169 cm^−1^, 1154 cm^−1^ and the in-plane bending at 960 cm^−1^ and 906 cm^−1^ [60,61,62,63]. DF shows n-alkanes with a band at 722 cm^−1^ [60,61,62,63]. TPO indicates a high intensity of tert-butyl groups with absorption bands at 1244 cm^-l^ and 1206 cm^−1^, which are weakly expressed in DF [61,62]. A high intensity of cyclohexene in TPO can be characterized by a band at 890 cm^−1^ and aligns with the presence of 1-methyl-4-(1-methylethyl)-cyclohexene detected in 2D-GC/MS analysis. Aromatic compounds can be identified in TPO and DF with valence and deformation vibrations at 3020 cm^−1^ and 1605 cm^−1^ [60,61]. Mono-aromatics such as alkyl benzenes can be assigned to the bands at 700 cm^−1^ and 740 cm^−1^. Di- and tri-substituted aromatics can be assigned to 770 cm^−1^, 806 cm^−1^, and 815 cm^−1^ [63]. Di-aromatics can be identified by the intermediate bands at 780 cm^−1^. The ring vibration of the aromatics in TPO can be associated with the two split bands at 1515 cm^−1^ and 1494 cm^−1^ [61,62]. The intensity of the aromatics bands, especially of di- and triaromatics, is considerably higher for TPO than for DF, indicating a higher concentration of the aromatic compounds, agreeing with the 2D-GC/MS and ^1^H-NMR results. Alkenes are also detected in the TPO spectrum, as indicated by C–H stretching at 3095 cm^−1^ and C=C stretching at 1640 cm^−1^ [61,62], whereas these features are absent in DF. These suggest that there are considerably less unsaturated compounds in DF than in TPO. These results confirm the findings from the 2D-GC/MS chromatograms and ^1^H-NMR-spectra. In addition, various oxygen-containing functional groups are evident in the TPO spectra. Carboxylic acids are indicated by a broad O-H band at 3395 cm^−1^ and a raised background down to 2800 cm^−1^. The presence of carboxylic acids can be confirmed by the C-O region between 990 cm^−1^ and 1120 cm^−1^ and the characteristic C=O band (1710 cm^−1^). However, these bands can also originate from other compounds like ethers, alcohols, carboxylic acids, carboxylate, and ketones [61,62,64]. In summary, the ATR-FTIR spectra clearly demonstrate that TPO contains significantly higher concentrations of aromatic and unsaturated compounds, as well as oxygenated species, which are largely absent in DF. These spectral differences underscore the distinct chemical compositions of the two substances.

#### 4.1.4. Further Chemical Analysis

The total acid number (TAN) (see Table 4) corresponds to the amount of potassium hydroxide (KOH) required to neutralize the acids present in one gram of fuel. DF exhibits an acid number of 0 mg g^−1^, indicating the absence of detectable acid compounds. With addition of TPO in DF, the TAN rises to 0.05 mg g^−1^ for DF99TPO1, 0.14 mg g^−1^ for DF95TPO5, 0.23 mg g^−1^ for DF90TPO10, and reaches a maximum of 2.08 mg g^−1^ for neat TPO. These findings corroborate the assumption derived especially from FTIR-spectroscopy, that acidic compounds are present in TPO. The iodine value serves as an indicator of the concentration of unsaturated compounds in olefines in a sample. The iodine value is calculated in accordance with DIN EN ISO 3961. The results show that the highest iodine value of 93 g 100 g^−1^ is observed for neat TPO, indicating a significant presence of unsaturated hydrocarbons. DF contains only a minimal fraction of unsaturated compounds, with an iodine value of 2.9 g 100 g^−1^. This value increases slightly with the addition of TPO: 3.1 g 100 g^−1^ for DF99TPO1, 3.7 g 100 g^−1^ for DF95TPO5, and 14 g 100 g^−1^ for DF90TPO10. These findings are consistent with the results obtained from ^1^H-NMR and FTIR-spectroscopy, confirming the presence of olefinic compounds in TPO. The aromatic content of TPO was quantified using High-Performance Liquid Chromatography (HPLC), according to DIN EN 12916. For this analysis, blends of DF and TPO were prepared. Based on linear regression (*R*^2^ = 0.9989) of the data presented for DF, DF99TPO1, DF95TPO5, and DF90TPO10 in Table 4, the total aromatic content of TPO was determined to be ~60 vol%. This result corroborates the findings of the current analyses and agrees with previously published studies reporting a high aromatic content in TPO [7,16,17,65,66].

In summary, the chemical composition of TPO differs from that of DF. TPO has a significantly higher proportion of aromatic and polyaromatic hydrocarbons. In addition, olefinic compounds as well as oxygen- and nitrogen-containing compounds, were identified in TPO compared to DF.

### 4.2. Characteristics of TPO and DF-TPO-Blends

To assess the suitability of TPO as a blend component in DF, the key values according to DIN EN 590-2022 for DF, TPO, and the blends are determined. The results of the analysis and the corresponding specific target ranges are summarized in Table 5. With a distillation start at 36 °C, TPO shows a remarkably low initial boiling temperature for fuels fractions. This is plausible, as the pyrolysis process can result in molecules with low boiling points and vapour pressure. In comparison, usually DF has a distillation range from 150 °C to 390 °C, with the DF used in this study showing a distillation start at 171 C.

On this basis, TPO is expected to also have a low flash point. The addition of 10 vol% TPO to DF results in a linear drop of the flash point to 53.1 °C below the specified limit of 55 C. As the TPO content increases, the start of distillation shifts to lower temperatures, reaching 157 °C at 10 vol% TPO. However, over the course of distillation, a divergence in temperature progression becomes apparent. 50 vol% distillate volume is reached at 273 °C for DF and at 275 °C for DF90TPO10. This trend, which other authors have also described, is that DF-TPO blends require a higher temperature than neat DF to distil the same volume percent, and this trend persists until the end of the distillation [67]. The end of the distillation is recorded at 390 °C at a distillate volume of 92 vol% for TPO exceeding the result for DF at 358 °C. This indicates that TPO contains higher-boiling components, compared to DF. DF-TPO blends adhere to the target values for DF at 90 vol% and at 95 vol%.

A solid, slightly porous residue remains in the distillation flask, which is also reflected in the carbon residue measurements. At 2.53 wt%, TPO has a higher value of coke residues compared with 0.02 wt% in DF, a consequence attributed to the polyaromatic content of 21.8 wt% in TPO. Whereas DF show a significantly lower content of 2.9 wt%, the blends show a linear increase to 4.7 wt% at 10 vol% TPO. The CFPP, which evaluates fuel filterability at low temperatures weather conditions, is also affected by the polyaromatic content. At low temperatures, polyaromatics tend to precipitate, causing an increase in the CFPP with higher TPO content. The density and viscosity of the fuels are strongly affected by their aromatic and polyaromatic composition. Since polyaromatic compounds possess higher densities than aliphatic species, an increased aromatic content results in a higher overall fuel density. TPO, characterized by a higher polyaromatic fraction, therefore exhibits a higher density and a lower viscosity compared to DF. Upon blending TPO with DF, the density of the blends increases proportionally with the TPO content, following an approximately linear correlation with TPO content. Overall, these results reveal the interdependence of characteristic values on the aromatic content. An increase in the polyaromatic content might not only affect the emission of hazardous substances and particles, posing challenges for modern engine and exhaust technologies, but also promote the formation of residues due to agglomeration during thermal processing [68,69].

Oxidation stability was evaluated by measuring the time required for the consumption of 1 bar of hydrogen after applying an initial pressure of 10 bar H_2_ to the fuel sample. Hydrogen reacts with unsaturated compounds present in the fuel, and the rate of consumption is proportional to their concentration. Thus, the higher the content of unsaturated compounds, the faster the pressure drops by 1 bar [70]. Due to the elevated concentration of unsaturated compounds formed during the pyrolysis process, TPO exhibits a low oxidation stability of 22 min, which is considerably lower than that of DF and the DF-TPO blends. The water content in TPO was determined to be 2053 mg kg^−1^, which significantly exceeds the values measured in DF and the DF-TPO blends. This elevated level is likely influenced by the presence of unsaturated compounds and free alkalis in TPO, which may interfere with standard water quantification methods [71].

According to current EU regulations, DF may contain no more than 10 ppm sulphur to limit the formation of sulphur oxides and sulfuric acid, which contribute to environmental pollution, catalyst degradation, and engine corrosion [72]. No visible corrosion was observed in the copper strip test for any fuel blend. The DF used in this study complied with specifications at 7.3 mg kg^−1^ sulphur. In contrast, TPO contained 1.16 wt% sulphur, far exceeding regulatory limits. Consequently, sulphur levels in DF–TPO blends increase linearly with TPO content. The high sulphur content in TPO is attributed to vulcanized rubber in tyres, which contains sulphur-based compounds [73,74]. Fuel lubricity was assessed using the High-Frequency Reciprocating Rig (HFRR), where lower wear scar diameters indicate better lubrication. DF and its blends showed relatively high wear values, while TPO exhibited superior lubricity, likely due to its elevated sulphur content, as sulphur compounds enhance lubricating properties.

In summary, the applicability of TPO and its blends with DF according to DIN EN 590 can be considered limited. The addition of 5 vol% TPO to DF results in parameters such as sulphur content, carbon residue, and CFPP exceeding the specified limits. At 10 vol% TPO, the flash point also falls outside the acceptable range, while oxidation stability and density approach the threshold values. Due to the significant changes observed with increasing TPO content, the quality of the base DF is of particular importance and should be determined in advance to assess the feasible blending ratio of TPO. Several parameters exhibit mathematical dependencies, allowing for the determination of the precise dosage that can be applied.

### 4.3. Swelling Behaviour of NBR and Its Influence on Mechanical Properties

#### 4.3.1. Mass and Volume Change in NBR After the Sorption

**Figure 4:**
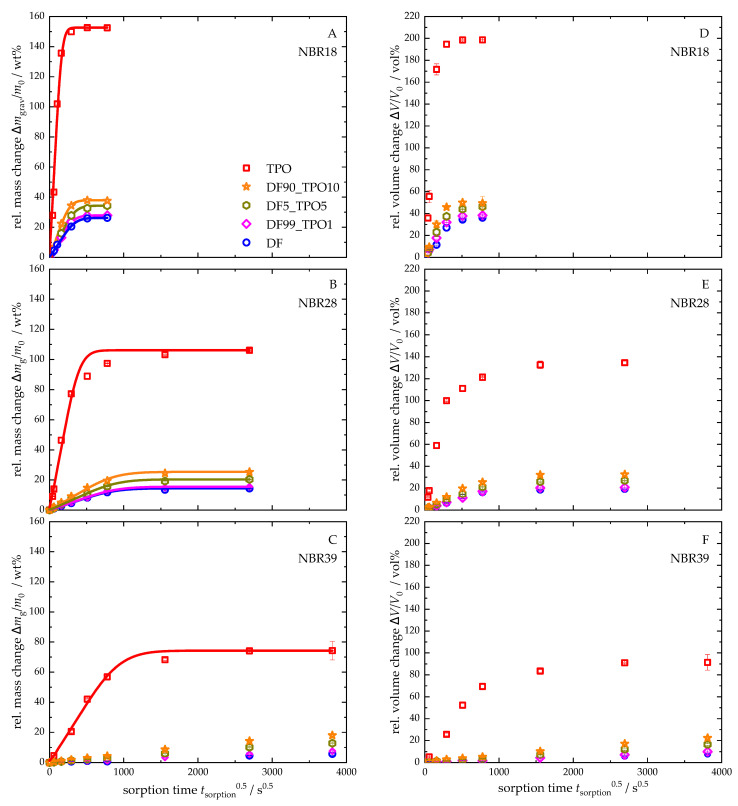
Relative mass and volume changes in NBR18 (**A**,**D**), NBR28 (**B**,**E**), and NBR39 (**C**,**F**) in DF, TPO and the mixtures with the ratios DF99TPO1, DF95TPO5, and DF90TPO10. The continuous lines show the calculated fit according to Equation (4). For NBR39 (**C**), the sorption curve could only be calculated for TPO, as these were in mass equilibrium.

Figure 4 presents the relative mass and volume changes in NBR18 (Figure 4A,D), NBR28 (Figure 4B,E), and NBR39 (Figure 4C,F), along with the corresponding sorption curves based on Equation (4), following storage in DF, TPO, and various DF-TPO blends. The mass changes are plotted as a function of the square root of time. The equilibrium mass uptake and the diffusion coefficients derived from these experiments are summarized in Table 6. For NBR39, equilibrium was not reached in DF or the DF-TPO blends within the experimental timeframe. As a result, no sorption curves and diffusion coefficient according to Equation (4) were calculated for these. All calculated sorption curves show an initial linear increase, fulfilling the requirement for applying Fick’s second diffusion law. The equilibrium mass uptake is lowest for DF across all elastomers, with values of 26 wt% for NBR18, 14 wt% for NBR28, and 6 wt% for NBR39. In contrast, TPO exposure results in the highest mass uptakes: 153 wt% (NBR18), 106 wt% (NBR28), and 74 wt% (NBR39), respectively. Identical trends can be observed for the relative volume changes in the NBR samples compared to the relative mass changes. With a volume increase of 199 vol% due to TPO, NBR18 exhibits an almost threefold expansion of the sample. NBR28 and NBR39 also exhibit a significant increase in volume with 134 vol% and 91 vol%, respectively, which underlines the pronounced influence of TPO on material behaviour.

**Figure 5:**
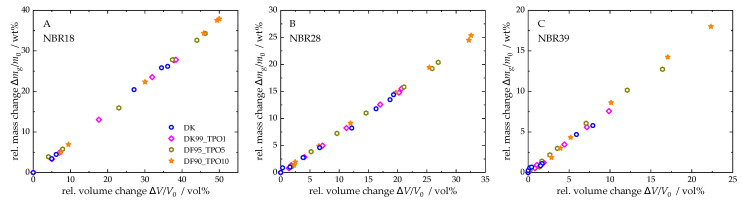
Relative mass change versus relative volume change in NBR18 (**A**), NBR28 (**B**), and NBR39 (**C**) for DF and TPO-DF blends.

To investigate the relationship between mass and volume of the NBR sample stored in TPO-DF blends, the relative mass change was plotted as a function of the relative volume change (see Figure 5). The data for pure TPO were excluded from the analysis, as their values are significantly higher than those of the blends, which would obscure a clear representation of the results. For NBR18, the blends exhibit a linear correlation between mass and volume changes. In contrast, NBR28 (for DF) and NBR39 (for DF and the blends) show a delayed volume expansion at the beginning of sorption, which is evident from the stage observed in the mass–volume relationship. With increasing TPO content, this delayed expansion diminishes. This behaviour can be attributed to the initially small mass uptake, during which the free volume of the NBR is filled before the material begins to swell. The addition of TPO leads to a more pronounced mass increase, thereby reducing this effect.

**Figure 6:**
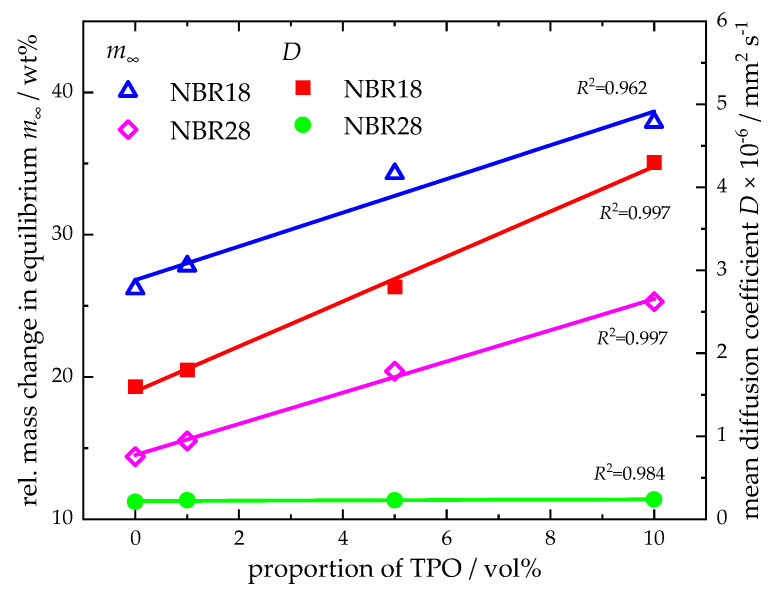
Relative equilibrium mass change and determined diffusion coefficients as a function of the volume fraction of TPO in DF. Linear fits with coefficient of determination (*R*^2^) are also given.

The diffusion coefficients follow the same trend as the equilibrium mass uptakes. For DF, the values are 1.6 × 10^−6^ mm^2^ s^−1^ (NBR18) and 0.21 × 10^−6^ mm^2^ s^−1^ (NBR28). For TPO results the diffusion coefficients of 11.8 × 10^−6^ mm^2^ s^−1^ (NBR18), 1.2 × 10^−6^ mm^2^ s^−1^ (NBR28), and 0.21 × 10^−6^ mm^2^ s^−1^ (NBR39). For the DF-TPO blends, the equilibrium mass uptake and the diffusion coefficients for the elastomers increase with rising TPO content. This relationship is clearly visible when plotting the equilibrium mass uptake and the mean diffusion coefficients against the TPO volume fraction in DF, as shown in Figure 6, where a linear correlation is observed.

The unusually high mass uptake observed for TPO can be attributed to its elevated aromatic content [25,75]. In addition to dispersion interactions, aromatic compounds in TPO also undergo π–π interactions with the acrylonitrile groups of the polymer matrix, which favours the sorption of these molecular classes [26]. Due to its higher acrylonitrile content, NBR39 exhibits stronger intramolecular interactions within the polymer chains, resulting in reduced free volume in the matrix. Consequently, NBR39 absorbs less fuel compared to NBR18 and NBR28. However, its greater affinity for the aromatic and polar compounds present in TPO results in a steeper initial slope in the sorption curves for NBR39, which means that equilibrium is reached faster when comparing TPO with DF. Additionally, low-molecular-weight aromatic compounds exhibit planar structures and less steric hindrance, which facilitates their diffusion into the polymer matrix more readily than larger, branched aliphatic molecules [25]. Since TPO contains polar compounds such as acids, enhanced swelling is expected due to the behaviour observed for fatty acid esters and alcohols in biodiesel [76,77,78]. These interactions reduce the intermolecular forces between the polymer chains, thereby increasing the matrix’s free volume and enlarging the effective surface area in contact with the liquid medium [79]. Consequently, the material’s surface tension can be affected, which reduces the resistance to sorption and facilitates further diffusion of TPO into the elastomeric network.

In summary, the pronounced and rapid diffusion of TPO into NBR, regardless of its high acrylonitrile content, typically selected for resistance to non-polar fuels, indicates that its use as a fuel in neat form is not recommended. However, depending on the blend ratio and the specific NBR formulation, the DF-TPO blends show mass uptakes and diffusion coefficients comparable to neat DF, suggesting that application in the form of blends appears possible.

#### 4.3.2. Temperature Dependence of the Diffusion Process of DF and TPO

**Figure 7:**
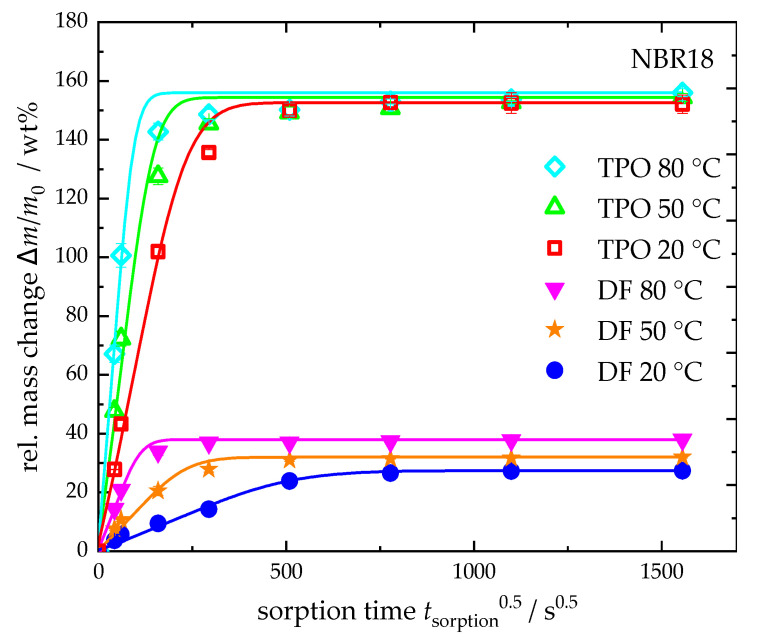
Relative mass change and sorption curves (Equation (4)) of NBR18 in neat DF and TPO at 20 °C, 50 °C and 80 °C.

In Figure 7, the sorption curves of neat TPO and DF at 20 °C, 50 °C, and 80 °C are plotted against the square root of time. For NBR18, the equilibrium mass uptake increases with temperature in DF, while it remains nearly constant for TPO. The diffusion coefficients, calculated using Equation (4), show a temperature-dependent increase for both fluids. In the case of DF, the diffusion coefficient rises by a factor of approximately 21 from 0.73 × 10^−6^ mm^2^ s^−1^ at 20 °C to 15.3 × 10^−6^ mm^2^ s^−1^ at 80 °C. For TPO, the diffusion coefficient increases by a factor of about six, from 3.43 × 10^−6^ mm^2^ s^−1^ to 21.8 × 10^−6^ mm^2^ s^−1^. The results are summarized in Table 7. It is well established that the diffusion of liquid media into elastomers increases with temperature. In a study by Blivernitz [26], the temperature dependence of model substances in NBR with 18 wt% acrylonitrile revealed that the equilibrium mass uptake of aliphatic compounds increases significantly with temperature, whereas aromatic compounds show either no change or only a slight increase. Consequently, the higher aliphatic content in DF results in a more pronounced temperature dependence. For DF-TPO blends, this implies that increasing the TPO content, thus raising the aromatic proportion, leads to a reduced temperature dependence of sorption behaviour.

**Figure 8:**
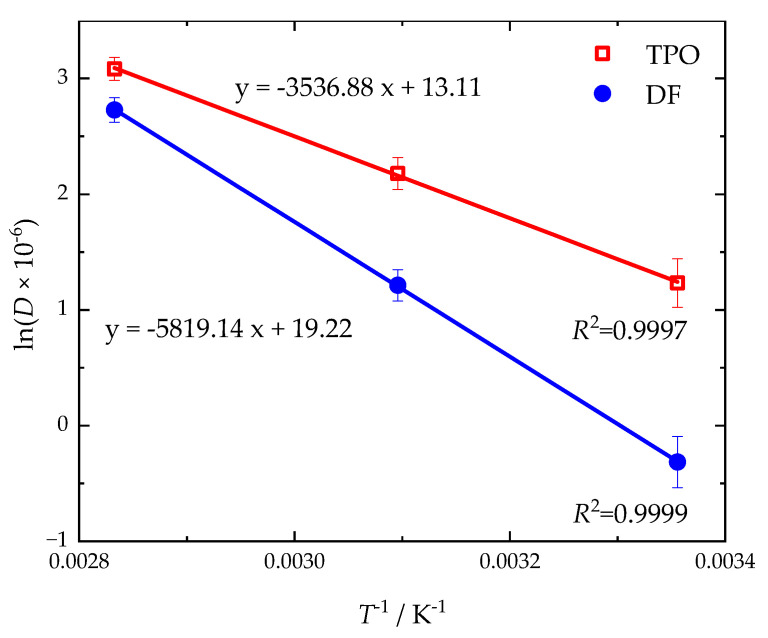
Arrhenius plot for the sorption of neat DF and TPO in NBR18. The coefficients of determination (*R*^2^) for the linear regression are also given.

The temperature-dependent diffusion coefficients can be used to determine the activation energy for diffusion according to Arrhenius (Equation (7)) [80]. In the Arrhenius plot (see Figure 8) the slopes (*E_A_*/*R*) of the linear regressions result in activation energies of 48 kJ mol^−1^ for DF and 29 kJ mol^−1^ for TPO.

(7) $$D=D_{0}\exp\left(-\frac{E_{A}}{RT}\right)\;\to \;\ln\left(D\right)=\ln\left(D_{0}\right)-\frac{E_{A}}{R}\;\frac{1}{T}$$

These results confirm the assumption that DF’s diffusion rate is more temperature-dependent than TPO. However, no statement can be made about the equilibrium absorption. It can be assumed that the diffusion properties of blends of TPO and DF are less dependent on temperature than neat DF. Applications of blends at elevated temperatures should therefore not show any deterioration of swelling behaviour with NBR.

In summary, a rise in temperature results in a faster rate of mass increase and higher total mass uptake. This observation is more evident in DF, demonstrating its stronger temperature dependence relative to TPO.

#### 4.3.3. Change in the Mechanical and Physical Properties of the NBR

**Figure 9:**
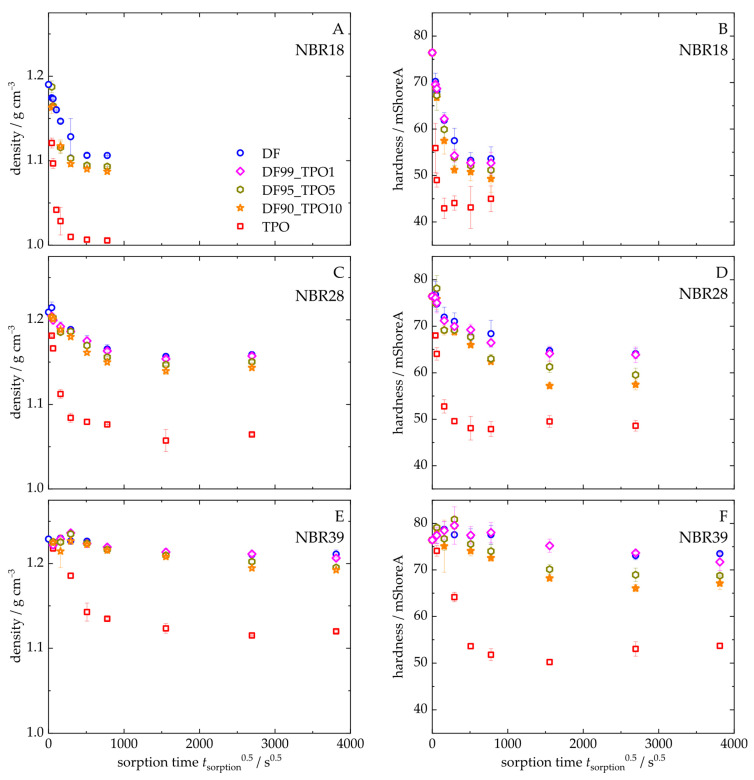
Density (**A**–**C**) and hardness (**D**–**F**) over sorption time of NBR18 (**A**,**D**), NBR28 (**B**,**E**), and NBR39 (**C**,**F**) after storage in DF, TPO, and the DF-TPO mixtures.

**Figure 10:**
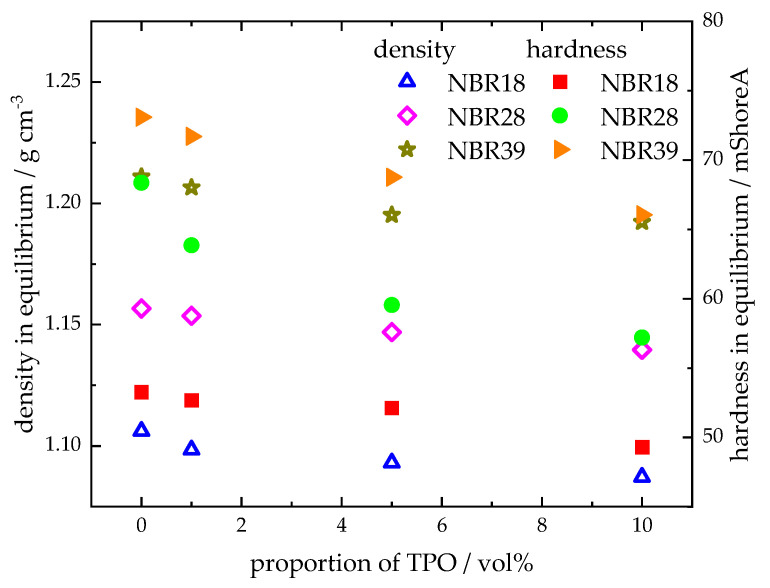
Density and hardness in equilibrium as a function of the volume fraction of TPO in DF.

Figure 9 illustrates the density and Shore A hardness of NBR18 (Figure 9A,D), NBR28 (Figure 9B,E), and NBR39 (Figure 9C,F) as a function of the square root of time. Both parameters decrease with increasing sorption time. The trend in density and hardness for NBR results from the mass uptake of TPO, as shown in Figure 4. During diffusion, both DF and TPO reduce the density of NBR, since the pristine material (ρ_NBR18_ = 1.19 g cm^−3^; ρ_NBR28_ = 1.21 g cm^−3^; ρ_NBR39_ = 1.23 g cm^−3^) has a higher density than TPO, ρ_TPO_ = 0.917 g cm^−3^, and DF, ρ_DF_ = 0.833 g cm^−3^. The decrease in hardness is attributable to the plasticizing effect of the absorbed fluids, which are stored between the polymer chains, weakening intermolecular interactions and thereby reducing the counterforce of the material. Blends containing up to 10 vol% TPO exhibit slightly lower density and hardness values compared to neat DF, indicating a measurable influence of TPO. As the acrylonitrile content increases, the reduction in equilibrium density and hardness upon exposure to DF, TPO, and their blends becomes less pronounced, following the order: NBR18 > NBR28 > NBR39. Figure 10 shows the equilibrium density and hardness values plotted against the TPO volume fraction in DF. The results indicate that, with increasing acrylonitrile content, the influence of the material to changes in density and hardness due to TPO in DF becomes more pronounced. The initial increase in density and hardness observed for NBR39 in Figure 9 is attributed to the slower diffusion of DF and the blends. In this case, the free volume within the polymer is filled before swelling occurs, which temporarily restricts chain mobility and leads to an increase in the mechanical counterforce of the material. Furthermore, it is observed that NBR28 and NBR39 reach equilibrium in hardness after approximately three days, which is earlier than the mass uptake shown in Figure 4. This discrepancy is explained by the fact that hardness measurements primarily reflect changes at the surface, where the medium penetrates first, while full diffusion into the sample core occurs later in accordance with Fick’s second law of diffusion.

**Figure 11:**
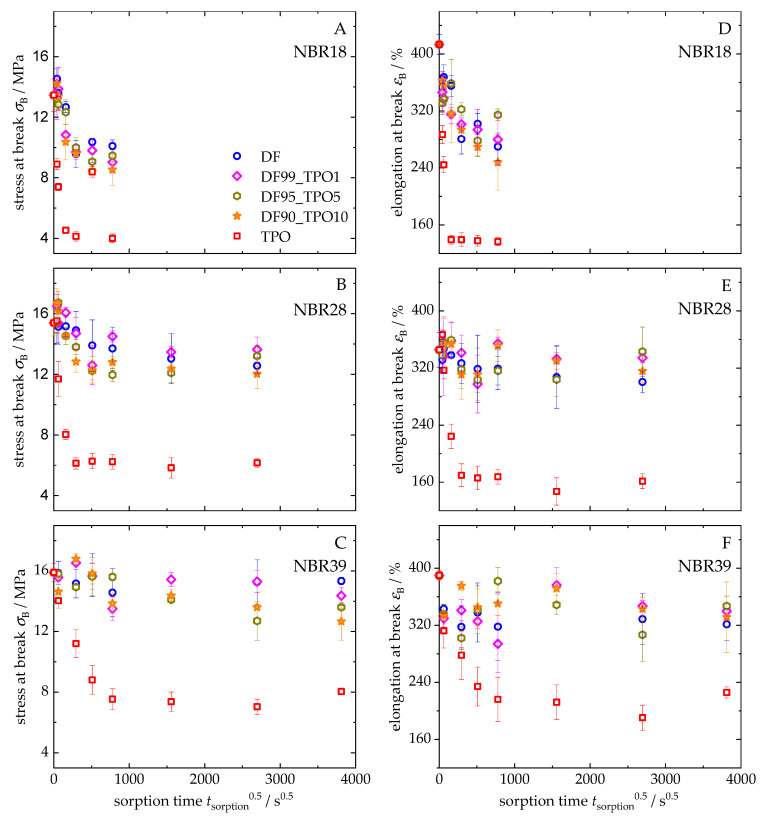
Stress (**A**–**C**) and elongation at break (**D**–**F**) over sorption time of NBR18 (**A**,**D**), NBR28 (**B**,**E**), and NBR39 (**C**,**F**) after storage in DF, TPO, and DF-TPO blends.

Figure 11 presents the development of tensile strength at break (Figure 11A–C) and elongation at break (Figure 11D–F) for NBR18 (Figure 11A,D), NBR28 (Figure 11B,E), and NBR39 (Figure 11C,F) as a function of the square root of time. For all elastomers, a decrease in both mechanical parameters is observed with increasing sorption time. The influence of TPO on the sorption behaviour is particularly pronounced, as its higher and more rapid mass uptake accelerates and intensifies the decrease in tensile strength and elongation at break compared to DF. NBR18 in TPO exhibits the strongest decline in mechanical properties, with tensile strength decreasing from 13.5 MPa to 4 MPa and elongation at break from 413% to 137%. In contrast, storage in DF and the blends results in significantly lower reductions. As the acrylonitrile content increases, the extent of mechanical characteristics decreases. NBR28 shows a decline from 15.4 MPa and 345% to 6 MPa and 161% while NBR39 decreases from 15.9 MPa and 390% to 8 MPa and 210% over three months (*t*^0.5^ = 2016 s^0.5^). For NBR28, a smaller reduction is observed, though the measurement scatter increases. In NBR39, a more pronounced reduction in tensile strength is evident even with 5 vol% and 10 vol% TPO in DF, with values decreasing to 15.3 MPa (DF) and 12.6 MPa (DF90TPO10). After six months of storage (*t*^0.5^ = 3810 s^0.5^), elongation at break for DF and its blends shows significant variation, ranging from 320% to 350%. The observed decrease in mechanical properties is directly related to the uptake of the liquid media. As previously described, sorption weakens the intermolecular interactions within the elastomer, facilitating chain mobility and thereby reducing strength under mechanical stress. The higher the mass uptake, the more severe the deterioration of mechanical integrity [81]. The particularly strong decrease caused by TPO can be predominantly attributed to its high aromatic content. This is explained by the specific interaction of aromatic compounds with the polar nitrile groups in the polymer, which disrupts the internal structure of the material.

In summary, none of the examined NBR types is suitable for long-term contact with neat TPO due to the pronounced loss in mechanical performance. However, blends with low TPO content show a decrease in mechanical characteristics comparable to neat DF, suggesting their potential suitability for application as a fuel.

## 5. Conclusions

In this study, tyre pyrolysis oil (TPO) was analyzed, and the characteristic properties of its blends with diesel fuel (DF) were determined. The sorption behaviour of these blends and the resulting changes in the mechanical properties of nitrile-butadiene rubber (NBR) were investigated. The objective of the study was to understand the TPO diffusion behaviour in elastomeric materials and to evaluate the feasibility of TPO as a fuel component.

Through detailed analysis using 2D-GC/MS, complementary analytical techniques such as infrared and NMR spectroscopy become more informative, allowing for the acquisition of more precise data. This combination of analytical methods enables a comprehensive and detailed understanding of the chemical composition. Chemical analysis revealed several notable differences between the composition of TPO and DF. Compared to DF, TPO contains the following:

- Higher monoaromatic and polyaromatic contents. This property makes TPO a potential blending component for aromatic-free synthetic fuels.
- Higher sulphur content. Desulfurization is required to reduce the sulphur concentration to within the target range. Alternatively, due to its high sulphur content and correspondingly low value in the high frequency reciprocating rig test, TPO could be considered as a blend component for lubricating oils.
- Higher proportions of olefinic compounds. Hydrogenation is necessary to saturate olefins and improve oxidation stability, thereby expanding the possible applications of both neat TPO and its blends with other fuels and oils.
- Larger amounts of low- and high-boiling components. Distillation of TPO to separate these fractions could produce a cut similar to DF, potentially lowering the polyaromatic content, as these are typically found in the high-boiling fraction, and raising the flash point by removing low-boiling compounds.

Without further chemical processing, the use of TPO in its raw form may present challenges. When blended at 1 vol% and 5 vol% with DF, most characteristic parameters remain within the target ranges specified by DIN EN 590, with the exception of sulphur content. However, under specific circumstances, such as emergencies or fuel shortages, use could be permitted according to StVZO § 70(4).

TPO exhibits strong affinity towards NBR, leading to rapid and substantial mass uptake and increasing volume during sorption. This pronounced swelling significantly reduces mechanical stability properties such as hardness, tensile strength, and elongation at break, thereby permanently affecting the viscoelastic behaviour of the elastomer. With increasing nitrile content, TPO uptake decreases, but equilibrium mass uptake is reached markedly faster for TPO than for DF. Increasing TPO content in DF results in proportionally higher absorption and diffusion coefficients, with a linear relationship observed between these parameters and the TPO volume fraction. Blends containing 1 vol% and 5 vol% TPO show no critical deviations in mass uptake, volume expansion, or mechanical properties compared to neat DF.

Further research is required to elucidate the interactions between individual TPO components and NBR. Analytical methods could be used to monitor the sorption of individual compounds, as unsaturated components or acids in TPO may react chemically with NBR, potentially altering its molecular structure and thus permanently impairing its mechanical performance.

## Figures and Tables

**Table 1:** Formulation of the three NBR elastomers with the specification in parts-per-hundred rubber (phr).

Ingredient	Concentration/phr		
Acrylonitrile content/wt%	18	28	39
Perbunan 1846	100		
Perbunan 2846		100	
Perbunan 3946			100
Carbon black (Typ N550)	60		
Di(2-propylheptyl) phthalate (DPHP)	20		
N-(4-Methylpentan-2-yl)-N-phenylbenzene-1,4-diamine (6-PPD)	2		
Zinc oxide	5		
Stearic acid	1		
Sulphur	2		
N-Cyclohexyl-2-benzothiazolylsulfenamide (CBS)	1.5		
Tetramethyl thiuram monosulfide (TMTM-80)	0.5		

**Table 3:** Intensity percentage in area% of the substance classes for DF and TPO.

Range	Substance Class	Intensity Percentage Area%	
		DF	TPO
1	Alkanes/Alkenes	74	8
2	Cycloalkanes	9	7
3	Aromatics	11	40
4	Indenes	4	12
5	Diaromatics	2	22
6	Polyaromatics	-	5
	Others *	≤1	6

* nitrogen-, oxygen-, and sulphur-containing compounds.

**Table 4:** Total acid number, iodine value, and aromatic content of DF, DF99TPO1, DF95TPO5, DF90TPO10, and TPO.

Characteristic	Units	DF	DF99TPO1	DF95TPO5	DF90TPO10	TPO
Total acid number	mg g^−1^	0	0.05	0.14	0.23	2.08
Iodine value	g 100 g^−1^	2.9	3.1	3.7	14	93
Aromatic content	wt%	24.1	24.4	26.0	27.7	~60 *

* with linear extrapolation (*R*^2^ = 0.9989) of the data of DF and DF-TPO blends.

**Table 5:** Characteristics of DF, DF99TPO1, DF95TPO5, DF90TPO10, and TPO according to DIN EN 590-2022.

Characteristic	Unit	Target	DF	DF99TPO1	DF95TPO5	DF90TPO10	TPO
Density	kg m^−3^	822.0–845.0	833.3	834.2	838.1	843.1	917.1
Viscosity	mm^2^ s^−1^	2.000–4.500	2.840	2.836	2.830	2.826	1.985
Polyaromatic content	wt%	≤8.0	2.9	3.0	3.8	4.7	21.8 *
Flash point	°C	>55	65.5	64.0	58.0	53.1	-
HFRR ^1^-Test	µm	≤460	420.5	359.5	222.5	209.5	175.5
CFPP ^2^	°C	≤−20	−25	−25	−13	−14	-
Cu corrosion	extent	1	1a	1a	1a	1a	1a
Oxidation stability	min	≥60	85	196	183	93	22
Water content	mg kg^−1^	≤200	37	30	60	40	2054
Coke residue	wt%	≤0.30	0.02	0.14	0.75	1.39	2.53
Ash	wt%	≤0.010	<0.001	0.001	0.001	0.001	-
Sulphur content	mg kg^−1^	≤10.0	7.3	134	648	1292	11,600 (1.16%)
Distillation curve							
Start	vol%		171.3	177.9	165.3	157.0	36.0
10% *v*/*v*			210.7	208.0	203.8	200.4	124.0
50% *v*/*v*			273.2	275.1	274.3	274.8	226.0
90% *v*/*v*		≤360	334.5	336.2	338.1	342.2	387.0
95% *v*/*v*		≤360	349.4	349.9	353.1	358.8	-
End			358.6	356.9	357.9	363.4	390.1

^1^ HFRR (high frequency reciprocating rig); ^2^ CFPP (cold filter plugging point), * with linear Extrapolation (*R*^2^ = 0.9989) of the data of DF and DF-TPO blends.

**Table 6:** Determined diffusion coefficients D and the relative equilibrium mass increases m_∞_ of NBR18, NBR28, and NBR39 after storage in DF, TPO, and the mixtures.

Fuel	NBR18		NBR28		NBR39	
	*m*_∞_	*D* × 10^−6^	*m_∞_*	*D* × 10^−6^	*m_∞_*	*D* × 10^−6^
	wt%	mm^2^ s^−1^	wt%	mm^2^ s^−1^	wt%	mm^2^ s^−1^
DF	26	1.6	14	0.21	-	-
DF99TPO1	28	1.8	16	0.23	-	-
DF95TPO5	34	2.8	20	0.23	-	-
DF90TPO10	38	4.3	25	0.24	-	-
TPO	153	11.8	106	1.2	74	0.21

**Table 7:** Mass change at equilibrium and determined diffusion coefficients of NBR18 in DF and TPO at 20 °C, 50 °C and 80 °C.

Sorption Temperature °C	DF		TPO	
	*m*_∞_	*D* × 10^−6^	*m_∞_*	*D* × 10^−6^
	wt%	mm^2^ s^−1^	wt%	mm^2^ s^−1^
20	27	0.73	153	3.43
50	32	3.36	154	8.83
80	38	15.3	156	21.8

## Data Availability

The original contributions presented in this study are included in the article. Further inquiries can be directed to the corresponding author(s).

## Abbreviations

The following abbreviations are used in this manuscript:

DF	Disel fuel
TPO	Tyre pyrolysis oil
NBR	Nitrile-butadiene rubber
NR	natural rubber
SBR	Styrene-butadiene rubber
BR	Butadiene rubber
phr	Parts-per-hundred rubber
DPHP	Di(2-propylheptyl)phthalate
6-PPD	N-(4-Methylpentan-2-yl)-N-phenylbenzene-1,4-diamine
TAN	Total acid number
HFRR	High frequency reciprocating rig
CFPP	cold filter plugging point
2D-GC/MS	Two-dimensional gas chromatography coupled to mass spectrometry
SPE	Solid phase extraction
GC/MS	Gas chromatography coupled to mass spectrometry
^1^H-NMR	1H-Nuclear magnetic resonance
FTIR	Fourier transform infrared
HPLC	High-Performance Liquid Chromatography
PAHs	Polyaromatic hydrocarbons
KOH	Potassium hydroxide
